# Development of an artificial intelligence based occupational noise induced hearing loss early warning system for mine workers

**DOI:** 10.3389/fnins.2024.1321357

**Published:** 2024-03-21

**Authors:** Milka C. I. Madahana, John E. D. Ekoru, Ben Sebothoma, Katijah Khoza-Shangase

**Affiliations:** ^1^School of Mining Engineering, University of the Witwatersrand, Johannesburg, South Africa; ^2^School of Electrical and Information Engineering, University of the Witwatersrand, Johannesburg, South Africa; ^3^Department of Audiology, University of the Witwatersrand, Johannesburg, South Africa

**Keywords:** occupational, noise-induced, hearing-loss, artificial intelligence, smart monitoring

## Abstract

**Introduction:**

Occupational Noise Induced Hearing Loss (ONIHL) is one of the most prevalent conditions among mine workers globally. This reality is due to mine workers being exposed to noise produced by heavy machinery, rock drilling, blasting, and so on. This condition can be compounded by the fact that mine workers often work in confined workspaces for extended periods of time, where little to no attenuation of noise occurs. The objective of this research work is to present a preliminary study of the development of a hearing loss, early monitoring system for mine workers.

**Methodology:**

The system consists of a smart watch and smart hearing muff equipped with sound sensors which collect noise intensity levels and the frequency of exposure. The collected information is transferred to a database where machine learning algorithms namely the logistic regression, support vector machines, decision tree and Random Forest Classifier are used to classify and cluster it into levels of priority. Feedback is then sent from the database to a mine worker smart watch based on priority level. In cases where the priority level is extreme, indicating high levels of noise, the smart watch vibrates to alert the miner. The developed system was tested in a mock mine environment consisting of a 67 metres tunnel located in the basement of a building whose roof top represents the “surface” of a mine. The mock-mine shape, size of the tunnel, steel-support infrastructure, and ventilation system are analogous to deep hard-rock mine. The wireless channel propagation of the mock-mine is statistically characterized in 2.4–2.5 GHz frequency band. Actual underground mine material was used to build the mock mine to ensure it mimics a real mine as close as possible. The system was tested by 50 participants both male and female ranging from ages of 18 to 60 years.

**Results and discussion:**

Preliminary results of the system show decision tree had the highest accuracy compared to the other algorithms used. It has an average testing accuracy of 91.25% and average training accuracy of 99.79%. The system also showed a good response level in terms of detection of noise input levels of exposure, transmission of the information to the data base and communication of recommendations to the miner. The developed system is still undergoing further refinements and testing prior to being tested in an actual mine.

## Introduction

Occupational noise-induced hearing loss (ONIHL) is a significant concern within the mining industry in South Africa (Khoza-Shangase, 2022), given the documented prevalence of high noise levels (Edwards et al., 2011). This prevalence of ONIHL is attributed to factors such as the nature of mining activities, the confined and reflective work environments, and the use of equipment in mines. These factors significantly increase the risk of exposure to hazardous noise levels, which are the primary cause of hearing problems among mine workers (Matetic, 2005; Strauss et al., 2012). Due to these factors, it has been estimated that one in four mine workers will develop ONIHL. As mine workers proceed to their mid-60’s, the incidence increases, with four out of five mine workers presenting with hearing impairment (NIOSH, 2023). To address this issue, South African mines implement hearing conservation programs (HCPs) aimed at protecting workers’ hearing health and minimizing the risk of ONIHL. The country has legislation and regulations that mines must adhere to such as the Occupational Health and Safety Act (OHSA) of 1993 (Republic of South Africa, 1995), along with its Noise-Induced Hearing Loss Regulations of 2003, which govern occupational health and safety in South Africa. These regulations set out specific requirements for noise exposure limits, hearing protection, audiometric testing, and the implementation of HCPs. The OHSA sets permissible noise exposure limits (NELs) for different industries and activities, including the mining industry. The regulations specify that the daily personal noise exposure level should not exceed 85 decibels (dB) for an eight-hour work shift.

Legislation and regulations, as part of the hierarchy of controls, also declare that, through risk assessments, employers are required to conduct noise risk assessments to determine the potential for hearing loss and identify areas where noise control measures are necessary. This involves measuring noise levels, evaluating exposure durations, and identifying high-risk areas or job tasks. At the same time, engineering controls measures should be in place to reduce noise levels at their source (NIOSH, 2023). This may involve using quieter machinery and equipment, isolating noisy equipment, or implementing sound insulation measures (Moroe and Khoza-Shangase, 2018a,b).

Additionally, employers are required to implement administrative controls to minimize workers’ exposure to excessive noise (Musiba, 2015). These controls may include limiting exposure time, scheduling rest breaks in quieter areas, and implementing job rotation to reduce individual exposure levels.

On the level of the employee and ranked as the last option on the hierarchy of controls, when engineering and administrative controls are insufficient to reduce noise levels to acceptable limits, employers are required to provide suitable hearing protection devices (HPDs) to their employees (NIOSH, 2023), HPDs that are properly selected, maintained, and used in accordance with regulations (Suter, 2002). Furthermore, employees must undergo regular audiometric testing as a crucial component of HCPs (Moroe et al., 2022). Employers are required to provide baseline audiograms for employees exposed to noise levels at or above the action level, followed by periodic audiometric monitoring to detect early signs of hearing loss (Moroe et al., 2022). Additionally, education and training should form part of HCPs where the goal is to raise employees’ and supervisors’ awareness about the risks of ONIHL (Moroe et al., 2018), proper use of HPDs (Ntlhakana et al., 2015), and the importance of complying with hearing conservation measures. For HCPs to be successful, legislation and regulations dictate that employers must maintain records of noise measurements, risk assessments, audiometric tests, and training provided to employees, and that these records should be readily available for inspection by relevant authorities (Amedofu and Fuente, 2008; Moroe N., 2020). Compliance with and enforcement of these regulations and legislation is the responsibility of the South African Department of Employment and Labour, which is responsible for enforcing occupational health and safety regulations, including those related to ONIHL.

Key points in South African legislation regulations, which comprehensively cover the hierarchy of controls including noise level limits, hearing conservation programs, engineering controls, education and training, monitoring and reporting are similar to those meant to be adhered to globally including in the Americas (Latin America, Canada, and the United States) and the rest of Africa (Arenas and Suter, 2014; Moroe et al., 2018). The main difference is the application and implementation of these, for example what each country’s defined values for permissible exposure limit (PEL) is, and if and how legislation enforcement occurs (Moroe et al., 2018). Where some countries ensure effective enforcement of regulations through inspections, penalties for non-compliance, and incentives for compliance; other countries do not (Moroe et al., 2018).

While HCPs in South Africa aim to address ONIHL, several challenges exist in their implementation (Moroe et al., 2018; Khoza-Shangase et al., 2020). Some documented common challenges associated with these programs include; lack of awareness and education among both employers and employees regarding the risks of ONIHL and the importance of hearing conservation measures (Moroe et al., 2018; Kanji et al., 2019). Inadequate and insufficient training and supervision regarding the implementation of HCPs, where employees and supervisors receive no or limited training on identifying noise hazards, selecting appropriate hearing protection, and conducting regular audiometric testing (Moroe and Khoza-Shangase, 2018a,b). Limited resources, including capacity versus demand challenges around audiologists in the country, leading to inadequate noise control measures, insufficient provision of HPDs, and limited access to audiometric testing facilities (Moroe et al., 2018; Pillay et al., 2020). Compliance issues around hearing conservation regulations where employers struggle to meet the requirements for noise measurements, risk assessments, audiometric testing, and recordkeeping, mostly due to some employers not prioritizing hearing conservation or attempting to cut costs by disregarding regulations (Khoza-Shangase, 2022). Linguistic, cultural and behavioral factors where, for example, attitudes towards wearing HPDs pose challenges; and the language used for training and education is incongruent with the employees (Moroe N., 2020). Effective enforcement and monitoring can be a challenge, influenced by insufficient resources and limited inspections by regulatory authorities resulting in inadequate enforcement of regulations and insufficient follow-up on non-compliant mines (Khoza-Shangase, 2022); and cumulative noise exposure and burden of disease (HIV/AIDS and TB) where some employees are exposed to high noise levels from multiple sources, both in their occupational and non-occupational environments, and suffer concurrent toxins exposure where they are on ototoxic treatments for HIV/AIDS and TB (Khoza-Shangase, 2022), thus increasing their risk of ONIHL and making it more challenging to control and mitigate the effects solely through workplace HCPs that do not take these factors into account. Addressing these challenges requires a multi-faceted approach that can be supported by the use of Internet of Things (IoT)-based hearing loss early monitoring systems as part of HCPs (Mardonova and Choi, 2018).

The main objective of this research work is to present a preliminary development of an AI based early monitoring system that integrates smart hearing protection with smart mine wearable watches. The developed system can provide significant benefits for mine workers as a form ONIHL early warning system. This system combines the capabilities of IoT devices, such as sensors and wearables, to monitor noise exposure levels and facilitate real-time monitoring and protection of the workers’ hearing when exposed to hazardous noise levels. By integrating IoT technology, smart hearing protection devices, and wearable watches, the current researchers aim to have a system that enables real-time monitoring, personalized protection, and early detection of hearing loss risks for mine workers. This system aims at enhancing worker safety, promoting proactive hearing health management, and contributing to a culture of prevention in the mining industry.

This early warning system includes numerous factors, for it to be efficient and successful, with positive outcomes for any HCP. Firstly, there has to be IoT sensors for noise monitoring that get strategically deployed in the mining environment to measure and monitor noise levels. These sensors can be placed in key areas or attached to equipment to capture accurate and real-time noise data. In the current study, these sensors are part of smart hearing muffs that transmit the data to a central monitoring system for analysis. The miners wear smart hearing protection devices (SHPDs) with smart watches which are also equipped with sensors. These SHPDs can have built-in noise sensors and connectivity capabilities to communicate with the central monitoring system. The SHPDs can adjust noise attenuation levels based on real-time noise exposure and provide workers with audio cues and alerts. The mineworkers are provided with wearable watches that act as a central hub for integrating various IoT devices and functionalities. These watches can connect to the SHPDs, IoT sensors, and other wearables, consolidating data and enabling real-time monitoring, communication, and alerts. Secondly, the early warning system must have real-time monitoring and feedback capabilities, where the IoT-based system continuously collects noise data from the sensors and SHPDs, transmitting it to the wearable watches. Mine workers can access real-time noise exposure information, receive alerts when noise levels exceed safe thresholds, and obtain feedback on their personal noise exposure. Thirdly, the system can allow for data analysis and insights development, where the collected data is analysed by the central monitoring system to identify patterns, trends, and potential risks. Machine learning algorithms can be employed to recognize patterns of noise exposure and detect early signs of hearing loss. The system can generate personalized reports and insights for individual workers and mine management. This can be done because the system has alert mechanisms, where if the system detects excessive noise levels or potential risks of hearing loss, it triggers alerts through the wearable watches. These alerts can be visual or auditory or vibrotactile, ensuring that mine workers are immediately aware of the hazards and can take necessary actions, such as adjusting their work practices or seeking quieter areas. Lastly, the system is set up to integrate with existing mine management systems and databases, allowing for seamless data sharing and accessibility. This integration facilitates comprehensive analysis, reporting, and decision-making processes related to hearing health and safety in the mining environment. Such an early warning system requires provision of comprehensive training to mine workers on using the IoT-based system, including the proper use of SHPDs and wearable watches. This includes conduction of awareness programs to educate workers about the importance of hearing protection and the benefits of the early monitoring system. These training sessions, and refresher courses, need to be conducted regularly to ensure effective and ongoing usage of the system. Ensuring that privacy and security considerations have been addressed is important as well. Implementation of robust privacy and security measures to protect worker data collected by the IoT devices, with compliance with data protection regulations (POPIA), secure data transmission protocols, and clear communication on data usage and privacy policies are essential to build trust among mine workers where such an IoT-based system is being used as part of HCPs (Ntlhakana et al., 2022). Another important consideration is the making sure that a robust maintenance plan to address issues related to IoT devices, wearables, and sensors is in place. Regular updates, calibration, and technical support are crucial to maintaining the reliability and accuracy of the system.

Once the AI-based ONIHL early warning system is in place, it can bring numerous value and benefits to both workers and the mining industry. This value and advantages include the following: Improved worker safety, early detection and intervention, personalised risk assessment, increased awareness and education, cost reduction, regulatory compliance, long-term data analysis, continuous monitoring, enhanced Occupational Health Programs (OHPs), and technological advancement and innovation. These benefits can be crucial in the context of mines, where noise is excessive.

## Background

Mining employees exposed to high noise levels often experience difficulty hearing high-frequency sounds initially (Edwards et al., 2010; Grobler et al., 2020). Regardless of age or gender, the measurement of hearing loss is typically assessed through percentage loss of hearing (PLH) and standard threshold shifts (STS) (Department of Labour, 2001; Department of Mineral Resources, 2016). PLH is determined by calculating the decline in hearing thresholds at specific frequencies (0.5, 1, 2, 3, and 4 kHz), and a baseline audiogram is established using this data (Department of Labour, 2001). South African hearing conservation practitioners employed this method for defining hearing loss for compensation purposes between 2001 and 2016 (Department of Labour, 2001). The STS method, based on the International Organization for Standardization (ISO) standard ISO1999:2013, considers an 8 dB decline as indicative of early ONIHL. Since 2016, South African mines have utilized the STS method to assess miners’ hearing, tracking STS deterioration as a precursor to hearing loss (Strauss et al., 2012; Grobler et al., 2020). In 2008, the Department of Mineral Resources and Energy (DMRE) established NIHL milestones for the mining industry, aiming to prevent hearing deterioration beyond 10 % in occupationally exposed individuals after December 2008 (Department of Minerals and Energy, 2008; Msiza, 2014). Despite efforts, hearing loss prevention was not entirely successful (Edwards and Kritzinger, 2012; Moroe and Khoza-Shangase, 2018a,b), leading to revised milestones in 2014, where no employee’s STS should exceed 10 dBHL from the baseline when averaged at 2000, 3000, and 4,000 Hz in one or both ears by December 2016 (MHSC, 2015; Moroe N. F., 2020). Therefore, STS became a prioritized metric for measuring hearing loss in miners.

Normal hearing is denoted as 0 dBHL (Chamber of Mines, 2016), and a STS is defined as an average shift in hearing threshold of 10 dBHL. While no hearing loss occurs at this stage, any shift greater than 10 dBHL should be reported, triggering further investigation and intervention (Chamber of Mines, 2016). A shift exceeding 25 dBHL for one or both ears indicates actual hearing loss, requiring diagnostic audiometry confirmation (Department of Mineral Resources, 2016). The use of STS to describe the hearing function of workers exposed to excessive noise levels has been a global practice since the early 2000s (Heyer et al., 2011; Masterson et al., 2015). The hearing loss prevention efforts in the South African mining industry, aligned with the NIHL 2016 milestones, now mirror those of developed countries like the United States. However, the efficacy of these interventions will only be assessed in 2024 (MHSC, 2015).

**Figure 1:**
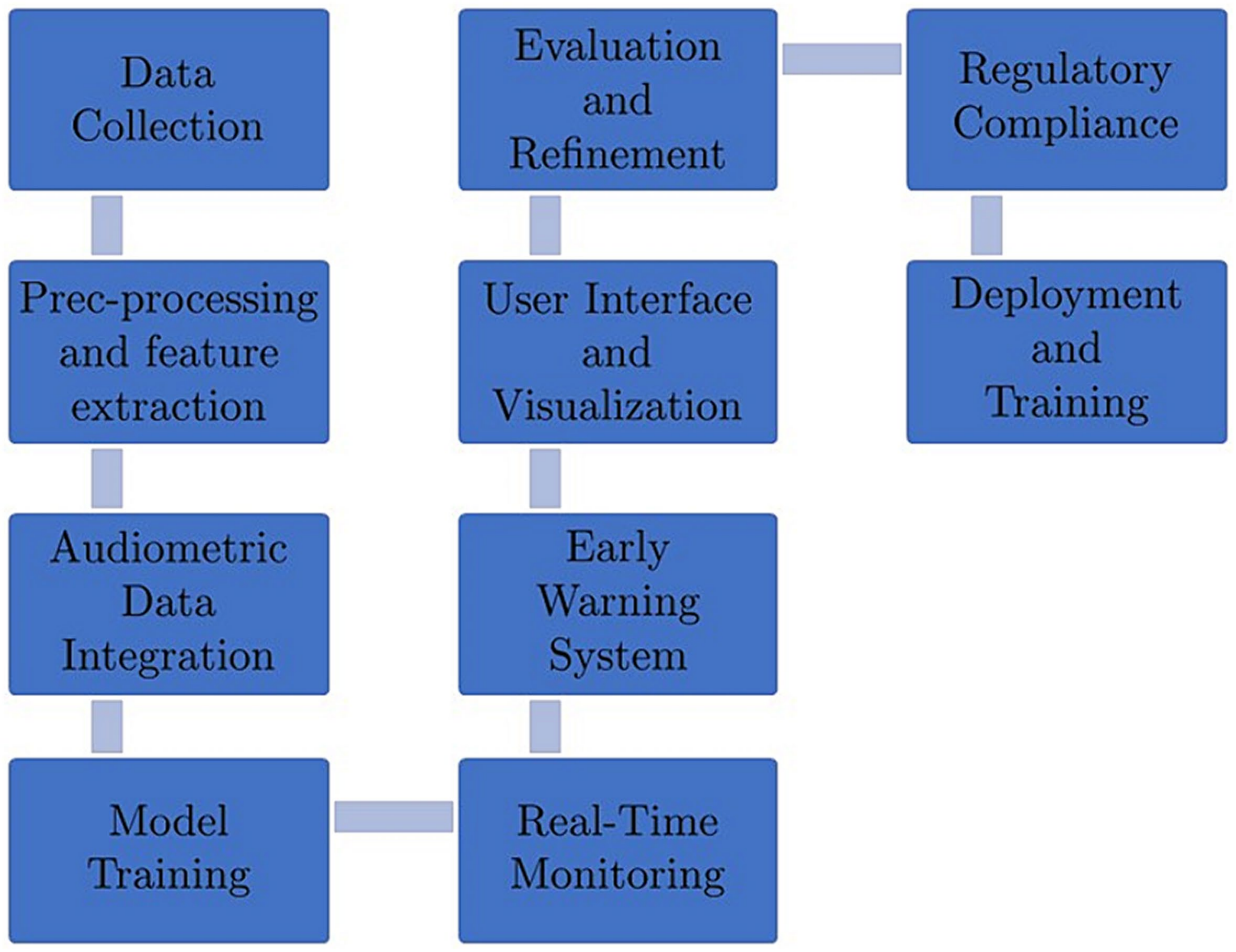
Development process for an AI-based ONIHL early warning system.

Developing an Artificial Intelligence (AI) based Occupational Noise Induced Hearing Loss (ONIHL) early warning system for mine workers can be a valuable initiative to safeguard their hearing health. Such a system can help identify potential risks and provide timely alerts to prevent or mitigate the harmful effects of noise exposure. The development process for such a system requires numerous steps depicted in Figure 1.

**Figure 2:**
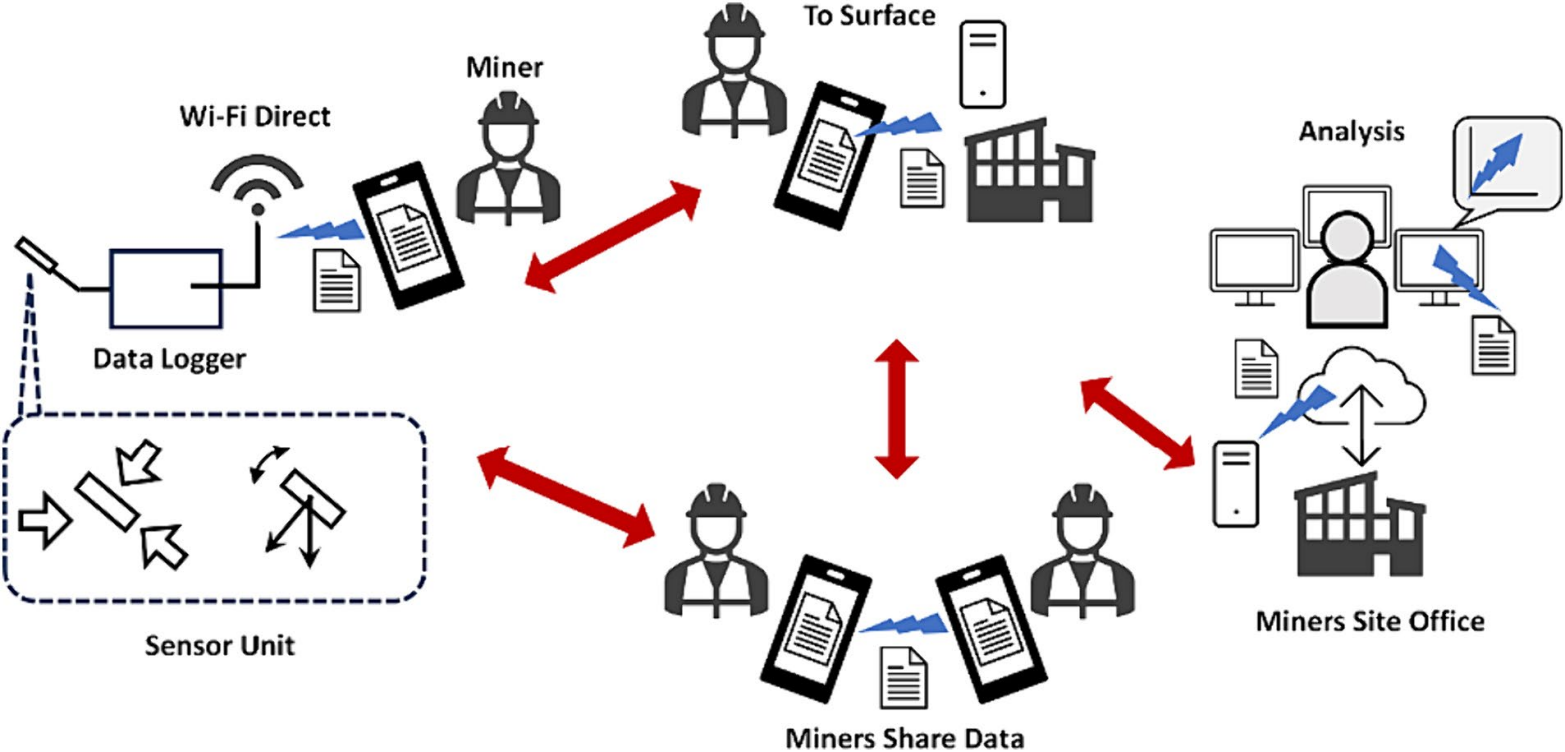
The data transmission diagram (Ikeda et al., 2021).

At present, various wired (Dohare et al., 2015; Ikeda et al., 2021; Kolade et al., 2021; Kolade and Cheng, 2021) and wireless communication technologies are available that meet the minimum mandatory criteria for the data broadcast speed and range to support remote mining operations and advanced monitoring systems. The data transmission diagram by Ikeda et al. (2021) and Figure 2.

The internet and WIFI technologies that are currently implemented in the mines ensure the efficient transmission and transfer of information. The transmission diagram in Figure 2 demonstrates how the smart technology is integrated with hearing protection.

## Materials and methods

Development of the AI based early warning system.

### Procedure

A smart system that continuously monitors noise levels in mine environments was developed. This system is made up of noise attenuation headphones, server-based AI algorithms and a smart watch. The headphones are equipped with sound sensors, and they collect information about the sound (sound intensity levels and the frequency) an individual mining employee is being exposed to. The dataset that contains the sound level of exposure for each mine worker is transmitted from the headphones to storage in a database. The collected data (sound intensity levels and frequency) is then fed into the trained AI model on the server.

To develop, train and test the AI subsystem of the smart system, a comprehensive dataset with various features is collected from various environments in a platinum mine. The features of interest in the collected data were noise level measurements, duration of exposure, corresponding audiometric test results, age, and gender. The data was cleaned and relevant features that can be used by the AI model such as duration of exposure, sound intensity of exposure and frequency, were extracted. The audiometric data were combined with the noise exposure data to establish the relationship between noise levels and hearing loss progression. This step was essential in training the AI models to recognize patterns and detect early signs of ONIHL. Machine learning techniques, random forest, support vector machines and logistic regression were utilized to train the AI model. For the AI subsystem, the target feature was the threshold shift of a miner worker defined as the average change in hearing of 10 decibels or more at speech frequencies (2,000–4,000 Hz) in both or one ear in comparison to the mine workers baseline audiogram. A 10-fold cross validation was ran with a split of 80 and 20% randomly shuffled training and testing set, respectively. The K means is used to cluster the mine workers and then using the threshold shift, the mine workers are classified the mine worker according to level of priority. A predicted threshold shift of less than 40 is viewed as low priority, between 40 and 60 is moderate priority, a threshold shift between 60 and 90 has a high priority and a threshold shift of greater than 90 has extreme priority. The various levels of priority are linked to various recommendations messages which are communicated to the mining employee via the smart watch. The low priority does not receive any messages while moderate priority receives a message to remind the mine workers to continue wearing their hearing protection correctly. The high and extreme priority receive a warning message and in addition to that a vibrotactile signal is triggered on the smart watch.

### Demographics and inclusion criteria

The initial training of the AI model required data. The data set used was obtained from a platinum mine in South Africa. The demographics of the dataset used to train the AI model is as follows: A total of 12,596 mine workers are in the platinum mine where this study was conducted. 11% of this mining population is female and 89% male. The age distribution indicates appropriate variation with 6,800 workers being younger than 40 years, 4,800 between ages 41 and 55, and 996 being between the ages of 55 and 65 years of age. The designed system targets occupations that are normally exposed to occupational noise for extended periods of time. Therefore, a dataset for 1,350 employees consisting of both male and female mining employees with ages ranging from 18 to 60 years old was used to train the AI model. This sample size for training the AI model was deemed adequate for reliable results as a good sample size is usually approximately 10% of the population, if this does not exceed 1,000 (Carmen and Betsy, 2007).

### General description of the subsystems of the developed early warning system

**Figure 3:**
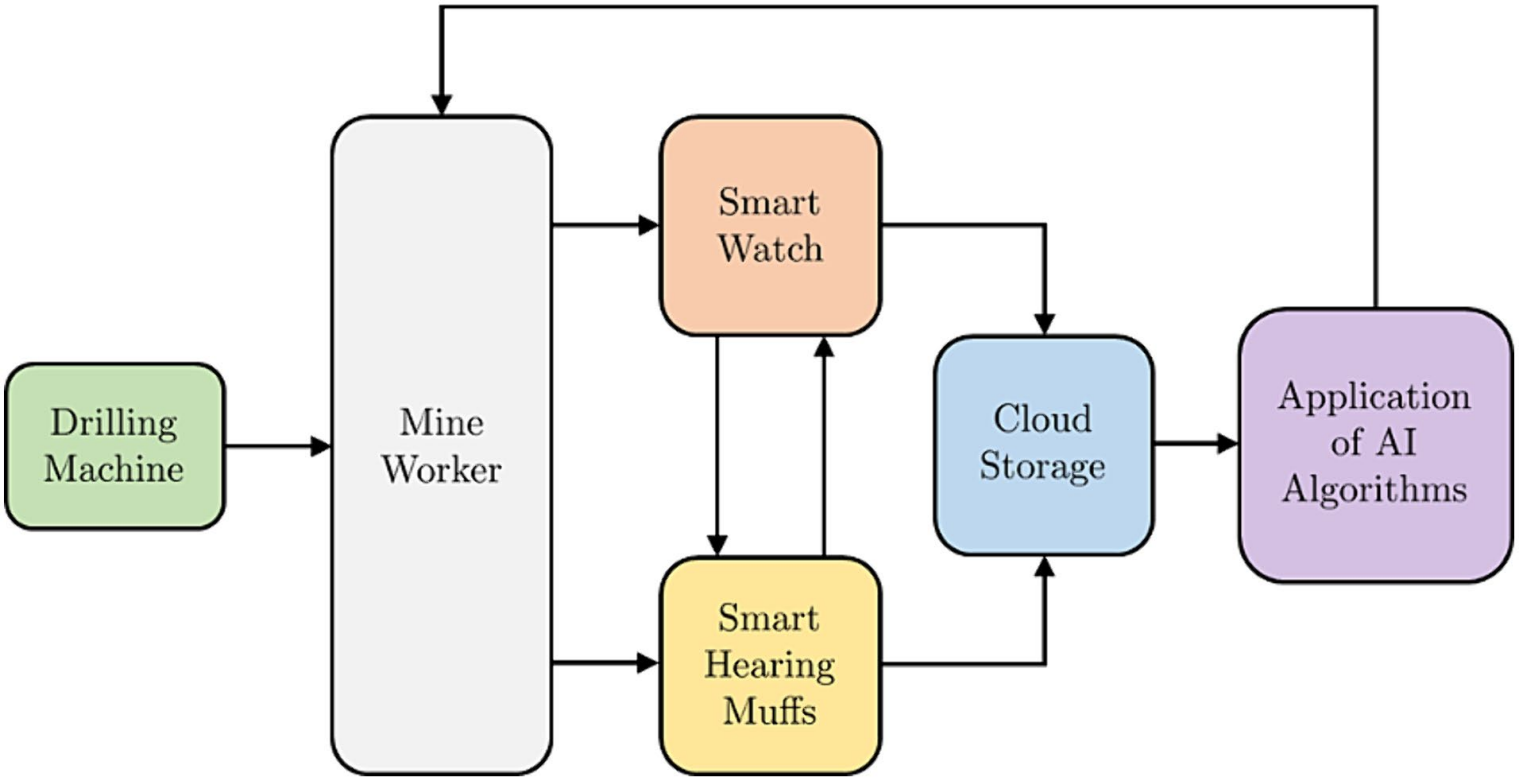
The block diagram of the ONIHL early warning and monitoring system.

**Figure 4:**
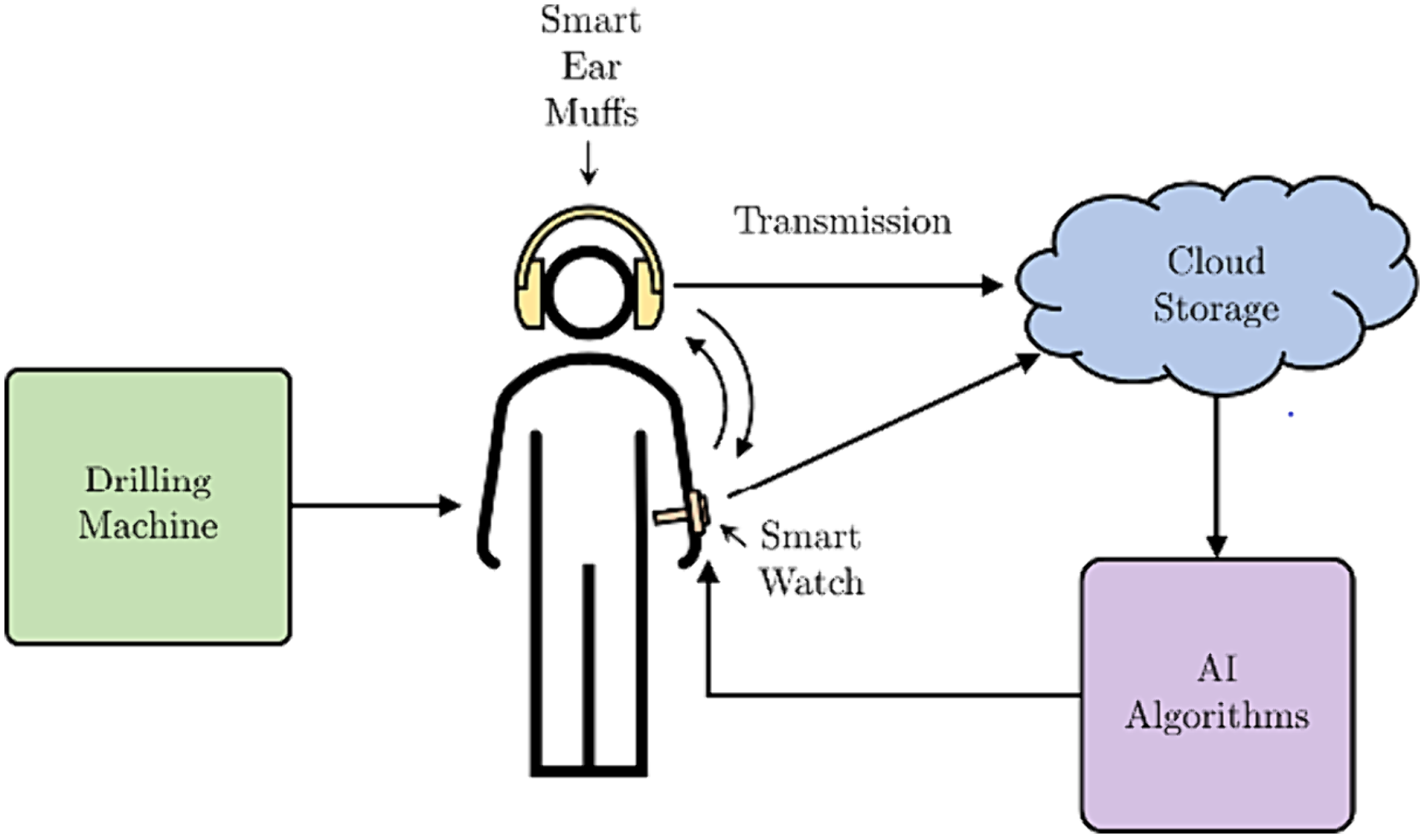
A pictorial representation ONIHL early warning and monitoring system.

Figures 3, 4 shows the block diagram and the pictorial representation of the Occupational Noise-Induced Hearing Loss and early warning system.

The mining employee is exposed to the level of noise the machine produces. A smart watch that capitalizes on the availability of WIFI and sensors in the mining environment is used to communicate with the hearing protection to provide mine workers with information about their surrounding and to enable communication. With the smart watch, the location of the individual mine worker can be established in real time by the mining administrators on the surface of the mine. This facilitates the ability of the administrators to check the conditions of the location, for example, the level of noise in the location where the mine worker is currently located. Personalised warning or recommendation messages can be sent to the mine worker. The important features the smart watch has for monitoring of the mine worker’s state of hearing are: Mine worker’s real-time location tracking, incident reporting and feedback-based communication. The mine workers can use the smart watch to report on incidences related to excessive noise that could be coming from faulty equipment or malfunctioning hearing protection. The integration of the system with Artificial intelligence permits for the real time automated early warning and recommendations alerts to be sent to the mine workers. The sound sensors set up within the mines provide essential information on areas within the mines with excessive noise that could be due to equipment failure. The mine worker can receive immediate alerts through the vibration of the smart watch. The smart watch applies IoT and can communicate with the hearing protection via Bluetooth technology. Several authors have made use of the ESP-32 as the basis for their smart-watch design (Volsa et al., 2022; Himi et al., 2023; Joseph, 2023; Puckett and Emil, 2023). The smart hearing protection can monitor real time noise levels using the sensors installed on it and cloud technology. The current levels of exposure to sound, which includes the intensity, and the frequency of exposure is collected by the hearing protection and transmitted to the cloud storage via a mobile app.

The mining administrators can conduct real time monitoring of the sound levels and the frequencies using the datasets. With this integrated system, the employees can be informed of their current levels of exposure at any time. With the integrated system, the mine worker can be informed whether the hearing protection is worn correctly or not. The mine worker can also be provided with warnings in the form of vibration of the smart watch which is integrated with the smart hearing device or other visual systems that can also be integrated into this system. The information collected from the smart watch and the hearing protection is stored in a robust data storage solution. To ensure control over the data and the application of the AI models, cloud storage is chosen. This type of storage ensures that the data can be recovered in case there is a problem on site in the mines. The cloud storage also requires little expertise for implementation with a few resources.

The AI subsystem is used in estimation of the mine worker’s threshold shift. The degree of priority is classified with the change in threshold shift. There are four classes of priority of threshold shift. These classes are low, moderate, high and extreme. The mining employee’s threshold shift is categorized and depending on the category recommendations are provided. The AI subsystem is also used to process the sound intensity patterns at particular frequencies and to provide the mine workers with recommendations of the actions they should take if necessary. Further details on the AI subsystems have already been published by the authors in previous works (Madahana et al., 2019a,b, 2020). The feedback loops allow for a two-way communication between the mine administrators on the surface and the mine employees. The feedback loops are from the mine administrator to the smart watch and from the smart watch to the hearing protection. These two systems can also be decoupled and in case the smart watch is not functioning, then the hearing muffs can still be used and in this case, the mine worker will depend on other visual warning systems in the mines that have been integrated with the system as a supplement.

### Implementation of the laboratory test rig

**Figure 5:**
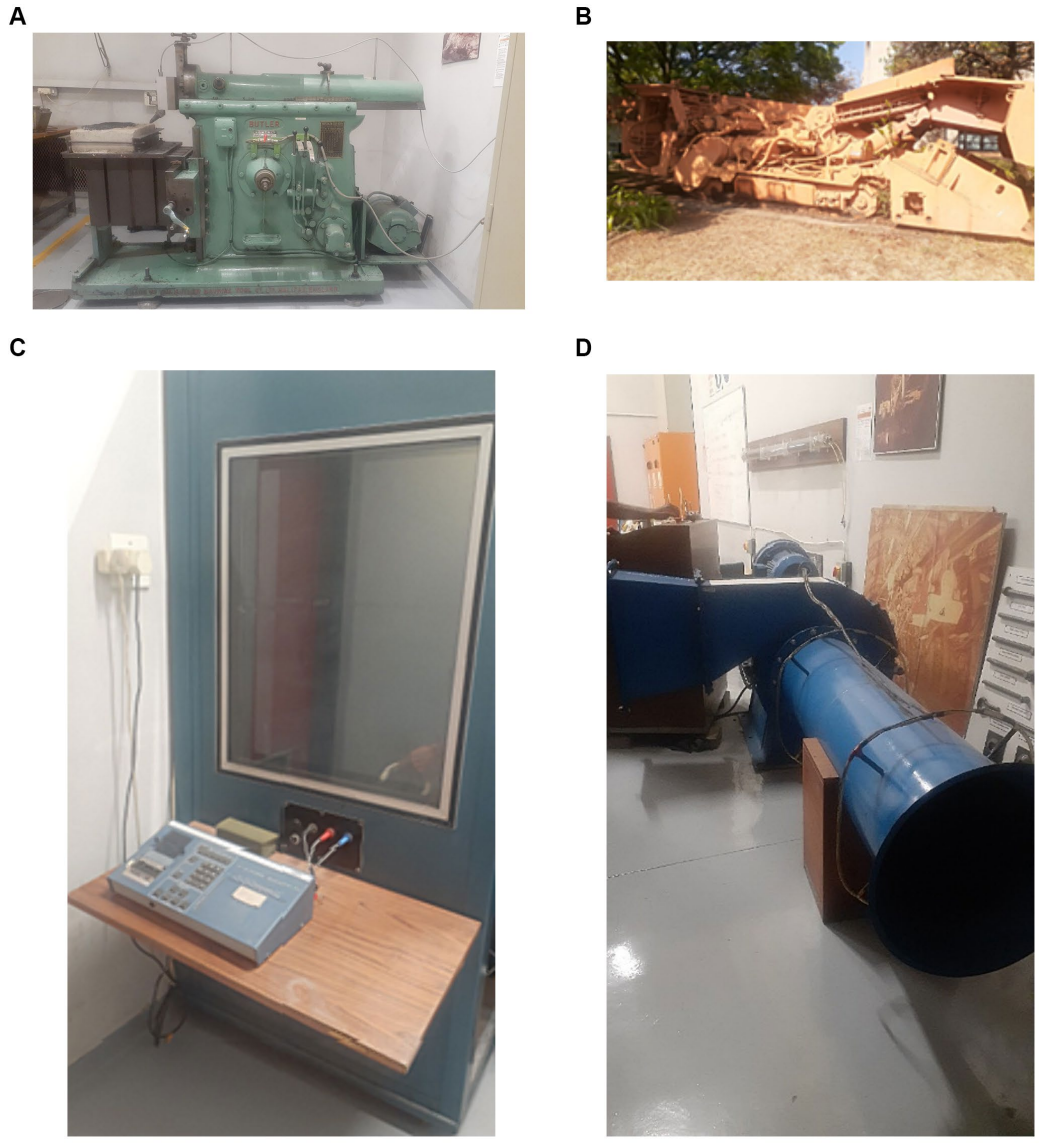
**(A)** The hydraulic shaping machines. **(B)** Shearing machine. **(C)** Variable direct current machines. **(D)** Psychometric booth.

**Figure 6:**
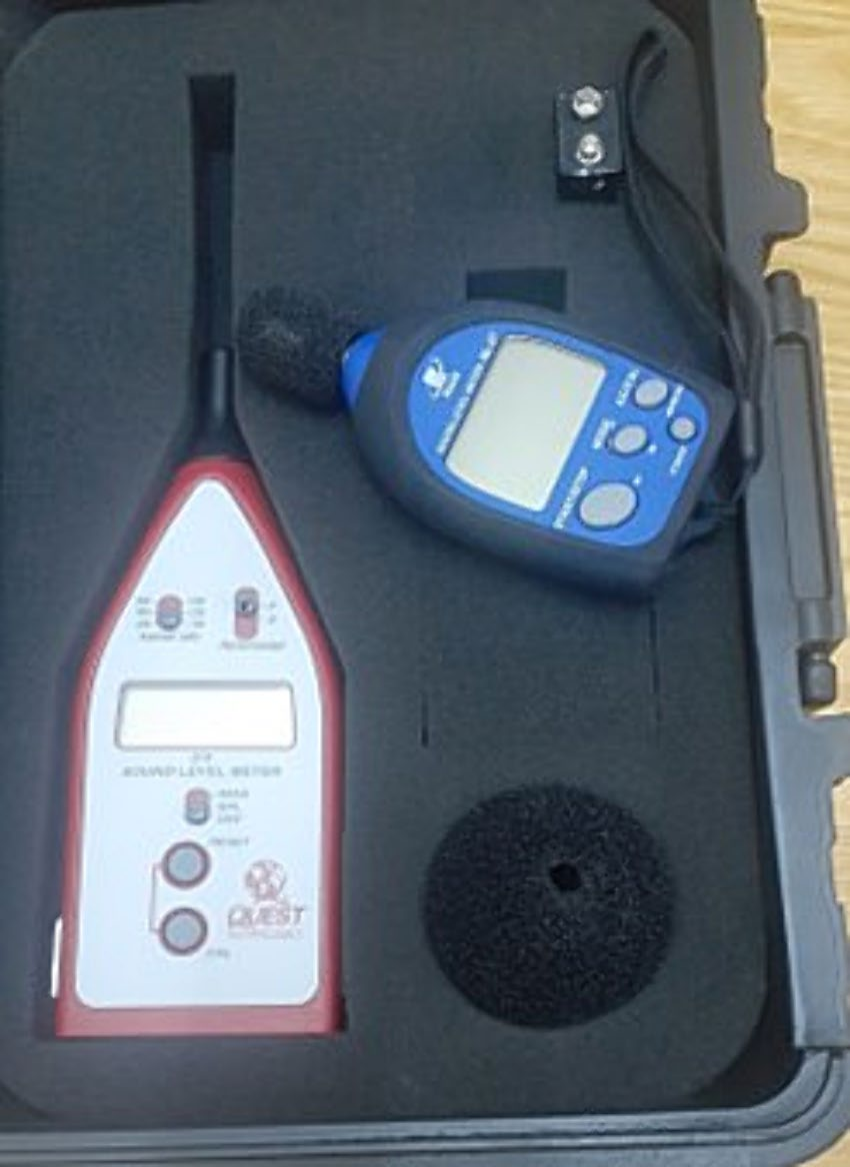
Sound level meter.

The laboratory test rig was built to test the proposed system. It consists of a smart watch, smart headsets, computer cluster, cloud storage, hydraulic shaping machine, Variable Direct Current (VDC) machine. The hydraulic shaping machine emits noise between 90 to 110 decibels depending on the various settings and activities. The Variable direct current emits a noise of 91.3–100.7 decibels. The system is connected as shown in Figures 3, 4, the drilling machine is replaced with the hydraulic shaping Machine and the VDC machine. The variable direct current machine shown in Figure 5C. The participants have their hearing checked in the psychometric booth shown in Figure 5D to ensure that their state of hearing health is known. The participant wears both the smart watch and the smart headset. The information obtained from the smart watch and the headset is transmitted via WIFI to a cloud storage. The information is then extracted from the cloud storage, AI models are applied to process the information and the appropriate recommendation is sent to the participant. The Participant tests the systems by moving 60 m away and thereafter, the distance is reduced until the participant is 0.5 m away from the machine. Different recommendation messages are sent to the participant smart watch at the various distance. The sound level metres in Figure 6 are used to measure the sound intensity of the machine. Figure 5A shows the shearing machine, which is simulated using hydraulic shaping machine, shown in Figure 5B. The preliminary integrated prototype is tested by allowing user to wear both the headphone and the smart watch, exposing them to noise at various decibels and frequency and observing the recommendations messages that are sent to the smart watch. One of the significant roles that audiologist plays during the testing of the integrated system is verification and validation of the system. The South African Mines usually have an audiologist who designs the Hearing Conservation Program for the mine. It is therefore imperative that audiologists be involved in the research, designing, testing and implementation of any system that would assist in minimizing the risks of ONIHL in the mines. The audiologist provides valuable feedback on the suitability of the integrated system and whether the user is wearing the hearing protection correctly.

The entire testing is conducted in the presence of audiologists to ensure that the participants are not exposed to any occupational noise and that the smart muffs provide sufficient attenuation.

**Figure 7:**
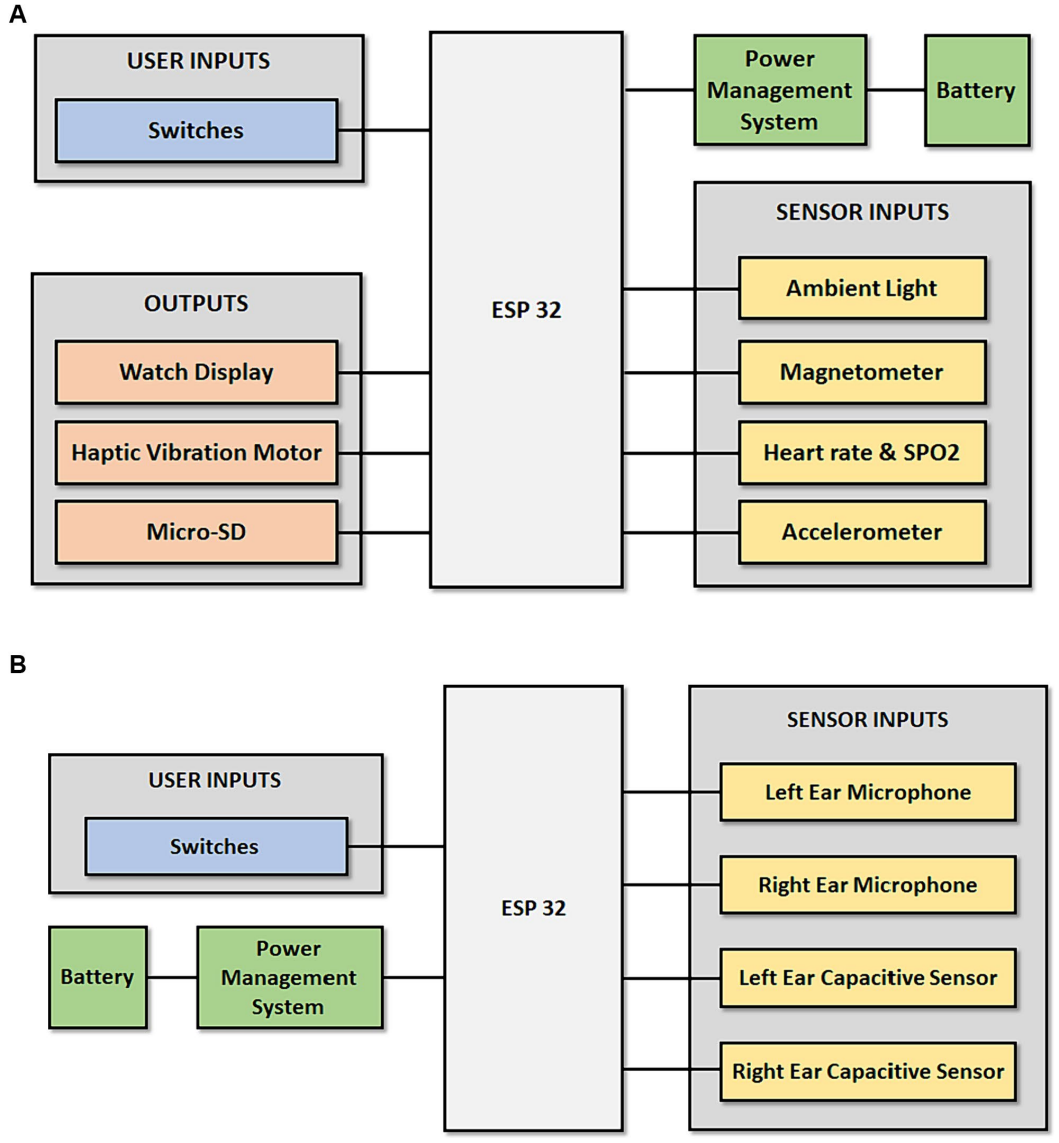
**(A)** Overall systems diagram indicating the subsystems in the ESP32 Smart watch. **(B)** Overall systems diagram indicating the subsystems in the ESP32 Smart Earmuffs.

Figure 7A shows the overall systems diagram of the Smart watch. The functioning of the smart watch is centred around the ESP32-WROOM-32 development board provides computational power as well as wireless internet and Bluetooth connectivity. The user inputs are switches which allow the mine worker to switch the watch on and off as well as toggle between various functions. The watch is powered by a Li-po battery, and a power management system is used to control the charging and discharging of the battery. Various sensor inputs are available to provide functionality during surface mining activities: (1) The ambient light sensor automatically adjusts the brightness of the screen and saves battery life. (2) The magnetometer is to be used for direction (compass). (3) Heart rate and Oxygen Saturation sensing for cardiovascular health. (4) The accelerometer for motion detection. During sub-surface mining, the magnetometer may be affected by the underground environment. The outputs of the smart watch are: (1) The Watch display which can be used to read time as well as notifications related to sound level and warnings related to NIHL due to environmental conditions. (2) The haptic vibration motor will vibrate during notifications as sent to the watch as well as when the mine worker is not wearing the hearing protection. (3) A micro-SD card is also available to log data.

Figure 7B shows the overall systems diagram of the Smart Earmuffs. Traditional earmuffs are equipped with additional sound sensors that can collect information from the environment. Similar to the smart watch, the ESP32-WROOM-32 development board provides computational power, wireless internet connection and Bluetooth connectivity. The user inputs are switches which allow the mine worker to switch the earmuffs on and off. The earmuffs are powered by a Li-po battery and a power management system is used to control the charging and discharging of the battery. The various sensor inputs are available to provide functionality during both surface and sub-surface mining activities: (1) The Microphones are used to pick up ambient sound to be used for a noise level meter that measures ambient sound in decibels. The noise level meter measurements are used to provide the mine worker with an instantaneous warning should the sound level reach dangerous limits. These warning messages appear on the smart watch accompanied by vibrations from the haptic vibration motor. In addition, the readings are sent wirelessly by the ESP32 for further processing in the cloud. (2) The capacitative sensors are used to detect whether the mineworker is correctly wearing the hearing protection. The messages are sent to the cloud and can be seen by administrators and warning messages are sent to the smart watch accompanied by vibration from the haptic vibration motor.

### Test environment

**Figure 8:**
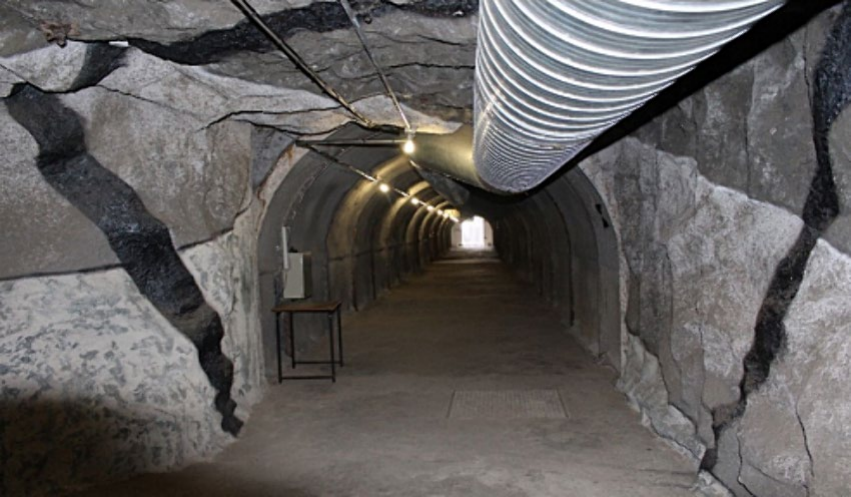
Wits mock mine tunnel.

Performing experiments in real underground environments is a rigorous process that requires permission from the mining stake holders. In addition to that, it can hinder normal operations from occurring while exposing researchers to unnecessary risks (Hussain et al., 2017). For rapid and repetitive testing of the developed prototype the Wits mock mine built under the Chamber of Mines building at the University of the Witwatersrand, Johannesburg (South Africa) was used. Some of the aspects of a mine that have previously been tested in this mock mine are and not limited to mine safety, tunnel economics, improved ventilation, energy savings and communication within a mine (Hussain et al., 2017). Comparable to actual mine, is made up of three sections: an arc shaped tunnel in the basement of the building, a stope panel and a vertical shaft. The tunnel is closed on one side and open on the other side. The roof of the mock mine represents the surface of the mine, and it is shallow from the open end and gets deeper towards the closed end. The mock-mine shape, size of the tunnel, steel-support infrastructure, and ventilation system are analogous to deep hard-rock mines. Actual underground mine material has been used to build the mock mine to ensure it mimics a real mine as close as possible. The mock mine is equipped with a weather station, asset management system, seismometer, crack meter, stress meter, asset management and video analytics system. The wireless channel propagation of the mock-mine is statistically characterized in 2.4–2.5 GHz frequency band (Zaman et al., 2018). Data from various systems is collected and displayed in the control room adjacent to the mock mine. The mock mine has an intelligent lamp room that prevents miners with inoperative equipment to enter the mine. It also has a rescue chamber, where all the miners can gather in case of a disaster. The tunnel for the mock mine is shown in Figure 8. The sample size used for the first stage of testing was 50 individuals made up of both male and female participants, whose ages ranged from 18 years to 60 years. The experimental protocols employed were approved by the ethics committee (The University of the Witwatersrand) and concur with the Helsinki Declaration. The study falls within the greater scope of another study titled “Feedback based estimation of Noise Induced Hearing Loss in the mines” and it has an ethical clearance number W-CBP-180305-01.

## Results and discussion

### Results

Table 1 shows the performance of the machine learning algorithm used. The Random forest classifier outperformed the other algorithms.

**Table 1:** Performance of the machine learning algorithms.

Model	Average training accuracy	Average testing accuracy
Logistic regression	74.56	77.25
Support vector machines	86.00	99.12
Decision tree	92.25	99.89
Random forest classifier	91.88	99.58

Table 2 shows the testing of the integrated system.

**Table 2:** Testing of the integrated system.

Distance of participant from machine (meters)	Priority level	Recommendation	Observation
Machine off	Low priority	None	No recommendations messages were received
60	low	None	No recommendations messages were received
50	low	None	No recommendations messages were received
40	low	Please wear your hearing protection	Successful SMS
30	Moderate	Hearing protection should be worn correctly	Successful SMS
20	Moderate	Hearing protection should be worn correctly	Successful SMS
10	Moderate	Hearing protection should be worn correctly	Successful SMS
5	High	Hearing protection should be worn correctly or step out of the section	Successful SMS
2	High	Hearing protection should be worn correctly or step out of the section	Successful
1	High	Hearing protection should be worn correctly or step out of the section	Successful
0.5	Extreme	Hearing protection should be worn correctly or step out of the section Vibration.	Successful SMS and vibration

## Discussion

In the context of the ONIHL, early warning and monitoring system for the mining industry, proactive and predictive approaches hold significant importance. By taking a proactive stance, the system identifies potential risks before they escalate, thus allowing for timely risk identification. It allows for the early detection of elevated noise levels and emerging patterns that could lead to hearing loss among mine workers. Early identification enables the implementation of preventive measures. These measures could include timely interventions, adjustments in work practices, or the use of enhanced PPE to minimize the risk of occupational noise-induced hearing loss. The predictive approach and the system’s predictive capabilities, driven by machine learning algorithms, allow for continuous monitoring of noise levels and associated factors. This ensures that any changes or trends in the working environment are promptly detected. Machine learning algorithms used in the developed system are trained to recognize patterns in the data. This includes identifying specific combinations of noise intensity, duration of exposure, and other variables that correlate with an increased risk of hearing loss. Predictive analytics help in forecasting potential issues based on these patterns. The predictive nature of the system enhances the alert mechanism. Instead of responding solely to current conditions, the system can anticipate future risks based on historical data, providing a more optimized and proactive alert system. Predictive analytics assist in the efficient allocation of resources. By forecasting when and where increased noise exposure is likely to occur, mine operators can deploy interventions strategically, focusing resources where they are most needed.

Combining proactive and predictive approaches allows for the development of comprehensive risk mitigation strategies. This involves not only addressing immediate concerns but also planning for long-term measures to reduce the overall risk of ONIHL in the mining environment. The goal is to enhance worker safety. Proactive measures prevent potential risks, while predictive analytics contribute to a more sophisticated and responsive safety infrastructure. This, in turn, minimizes the likelihood of ONIHL incidents. Being proactive in identifying and addressing risks ensures that the developed system aligns with regulatory standards. This is crucial for maintaining compliance with occupational health and safety guidelines specific to noise exposure in mining operations. By adopting proactive and predictive approaches, the ONIHL early warning and monitoring system aims for a lasting impact. Continuous evaluation, refinement, and adherence to safety protocols contribute to sustained worker well-being over the long term.

An alert mechanism that triggers warnings when the AI model detects excessive noise levels or predicts an increased risk of ONIHL for mine workers was implemented. These alerts can be sent to the workers, supervisors, or safety officers through visual or auditory means. Integrated smart hearing protection and wearable mining watches can contribute to hearing loss prevention as they could be categorized as PPE and administration in the hierarchy of controls. These form part of preventative audiology efforts where the focus is on *preventive care* rather than *compensatory care.* This preventive goal is achieved when the smart watch and the hearing protection work collaboratively to ensure preservation of hearing among mine workers by sending alerts and recommendation messages regarding the work context as well as the miner’s state of hearing.

## Recommendations and conclusions

In summary, the importance of proactive and predictive approaches, as in the proposed, lies in their ability to prevent, identify, and address risks systematically, fostering a safer and healthier working environment for mine workers. The ONIHL early warning and monitoring system employs a holistic approach, integrating advanced technologies, machine learning, and real-time monitoring to effectively address the risk of ONIHL in the mining industry. Bearing the above in mind, several steps remain to be completed in future. Firstly, evaluation and refinement of the system still needs to be done. The performance of the AI model and the effectiveness of the early warning system need to be continuously evaluated. In this process, feedback from mine workers and stakeholders will be collected to identify areas for improvement and refine the system accordingly. Secondly, regulatory compliance needs to be ensured. The developed system requires the researchers to ensure that it aligns with relevant safety regulations and standards for noise exposure in mining operations. Compliance with occupational health and safety guidelines is crucial to ensure the well-being of workers. Lastly, deployment and training of the system is yet to be performed. The system still needs to be deployed in mine sites and adequate training be provided to workers and supervisors on how to interpret and respond to the warnings. Regular training sessions and awareness programs can help promote a safety-conscious culture. The development design and preliminary implementation of a test prototype for a ONIHL early warning and monitoring system has been presented. This system will play a fundamental role in ensuring that the risks of ONIHL in the South African mines is minimized or mitigated. For this system to be work efficiently, mine workers will have to be trained on the correct ways to wear the smart watches and the hearing protection.

## Data availability statement

The original contributions presented in the study are included in the article/supplementary materials, further inquiries can be directed to the corresponding author.

## Ethics statement

The studies involving humans were approved by University of the Witwatersrand ethics committee. The studies were conducted in accordance with the local legislation and institutional requirements. Written informed consent for participation was not required from the participants or the participants’ legal guardians/next of kin in accordance with the national legislation and institutional requirements.

## Author contributions

MM: Conceptualization, Data curation, Formal analysis, Funding acquisition, Investigation, Methodology, Project administration, Software, Validation, Writing – original draft. JE: Data curation, Investigation, Methodology, Software, Validation, Writing – review & editing. BS: Formal analysis, Investigation, Validation, Writing – review & editing. KK-S: Conceptualization, Formal analysis, Investigation, Methodology, Supervision, Validation, Writing – review & editing.
